# Author Correction: Microscale Schottky superlubric generator with high direct-current density and ultralong life

**DOI:** 10.1038/s41467-021-23563-5

**Published:** 2021-05-14

**Authors:** Xuanyu Huang, Xiaojian Xiang, Jinhui Nie, Deli Peng, Fuwei Yang, Zhanghui Wu, Haiyang Jiang, Zhiping Xu, Quanshui Zheng

**Affiliations:** 1 Center for Nano and Micro Mechanics, Tsinghua University, Beijing, 100084 China; 2 Department of Mechanical Engineering, Tsinghua University, Beijing, 100084 China; 3 State Key Lab of Tribology, Tsinghua University, Beijing, 10084 China; 4 Department of Engineering Mechanics, Tsinghua University, Beijing, 100084 China; 5 Institute of Superlubricity Technology, Research Institute of Tsinghua University in Shenzhen, Shenzhen, 518057 China

**Keywords:** Materials chemistry, Devices for energy harvesting, Mechanical properties

Correction to: *Nature Communications* 10.1038/s41467-021-22371-1, published online 15 April 2021.

This article contains an error in equation (6) in the original manuscript where one symbol was wrong. The corrected version is:

$$\begin{array}{c}\begin{array}{c}\begin{array}{c}\vec{{{\boldsymbol{J}}}_{{\bf{n}}}}=-{qn}{\mu }_{{\rm{n}}}\nabla V+q{D}_{{\rm{n}}}\nabla n,\\ \vec{{{\boldsymbol{J}}}_{{\bf{p}}}}=-qp{\mu }_{{\rm{p}}}\nabla V-q{D}_{{\rm{p}}}\nabla p,\end{array}\\ \begin{array}{c}\nabla \cdot \vec{{{\boldsymbol{J}}}_{{\bf{n}}}}=q\frac{\partial n}{\partial t},\\ \nabla \cdot \vec{{{\boldsymbol{J}}}_{{\bf{p}}}}=-q\frac{\partial p}{\partial t},\end{array}\end{array} \qquad \left(6\right)\end{array}$$

The original version of this Article (main manuscript and Supplementary Information) contained an error in the spelling of the author Jinhui Nie, which was incorrectly given as Jinghui Nie.

## Supplementary information

Supplementary information

