# Supplementary material for: Microscale Schottky superlubric generator with high direct-current density and ultralong life
Source: Nat Commun. 2021 Apr 15;12:2268. doi: 10.1038/s41467-021-22371-1 (PMC8050059; doi:10.1038/s41467-021-22371-1)
Supplement: Supplementary file 1 — Supplementary Information [file 41467_2021_22371_MOESM1_ESM.pdf]

Microscale Schottky superlubric generator with high direct-current density and ultralong life

Xuanyu Huang[1,2,3], Xiaojian Xiang[1,4,5], Jinhui Nie[1,4,5], Deli Peng[1,4], Fuwei Yang[1,4], Zhanghui Wu[1,4], Haiyang Jiang[1,5], Zhiping Xu[4], Quanshui Zheng[1,2,3,4,5*]

[1]Center for Nano and Micro Mechanics, Tsinghua University, Beijing 100084, China;

[2]Department of Mechanical Engineering, Tsinghua University, Beijing 100084, China;

[3]State Key Lab of Tribology, Tsinghua University, Beijing 10084, China;

[4]Department of Engineering Mechanics, Tsinghua University, Beijing 100084, China;

[5]Institute of Superlubricity Technology, Research Institute of Tsinghua University in Shenzhen, Shenzhen 518057, China.

*E-mail: zhengqs@tsinghua.edu.cn

(Supplemental Imformation)

Content

1.The fabrication process of graphite mesa with Au film and n-Si 3

2.The preparation of S-SLG 4

2.1 The transfer process of graphite flake 4

2.2 The work function measurement of n-Si and HOPG 5

3.Friction and current measurements of AFM system 7

3.1 Calibration of AFM tip 7

3.2 Noise current measurement of AFM system 8

3.3 Current decay caused by graphite flake delamination 9

4.Open-circuit voltage and power measurement 11

4.1 Noise current and voltage measurement 12

4.2 The open-circuit voltage and short-circuit current waveform under different normal force 12

4.3 The I-V characteristics of graphite/n-Si interface under different normal force 13

4.4 The open-circuit voltage measured by null method 15

5.The contact area calculation of AFM tip/n-Si ordinary S-G 17

6.Verification of the SSL state in graphite/n-Si S-SLG 19

6.1 Surface characterization method of tribological experiment 19

6.2 The graphite defects resolution estimation of Raman measurement 23

7.The tunnelling probability of electron through Schottky barrier in S-SLG 23

8.The quasi-static simulation of DLED mechanism 24

8.1 The setup of simulation 24

8.2 The simulation process 26

8.3 The unbalance electric potential and electric field distribution 28

8.4 The estimation of unbalanced electron drift motion characteristic time 30

9.The discussion and classification of all reported ordinary S-G 31

1. The fabrication process of graphite mesa with Au film and n-Si

In order to get graphite flake, we firstly fabricated square graphite mesa arrays with Au film on highly ordered pyrolytic graphite (HOPG, ZYB grade (Brucker) [1]). In the fabrication process as shown in Supplementary Fig. 1a, we firstly spun on a double layer photoresist LOR 1A (100 nm)/ZEP (400 nm) on the fresh cleavage surface of the HOPG (i), and then removed the photoresist of the mesa array area by electron beam lithography (ii). Secondly, we grew the Au array film with thickness of 100nm through electron beam evaporation (includes the 10nm Cr as adhesion layer) (iii) and lift-off (iv) process. Lastly, we used metal as a mask to obtain graphite mesa with Au film by reactive ion etching (oxygen ions) process (v), where the etching depth is 2.5 µm.

In order to show the effect of the fabricated graphite mesa with Au film, we carried out a series of characterizations, where the optical images of fabricated graphite mesa with Au film validated by optical microscopy (HiRox KH-3000) are shown in Supplementary Fig. 1b, the brighter area in the images is the protruding mesa. And the three-dimensional white light interference image of graphite mesa with Au film is shown in Supplementary Fig. 1c, where the bottom image is a sectional view of a graphite mesa, the height of the graphite mesa is 2.6 µm.

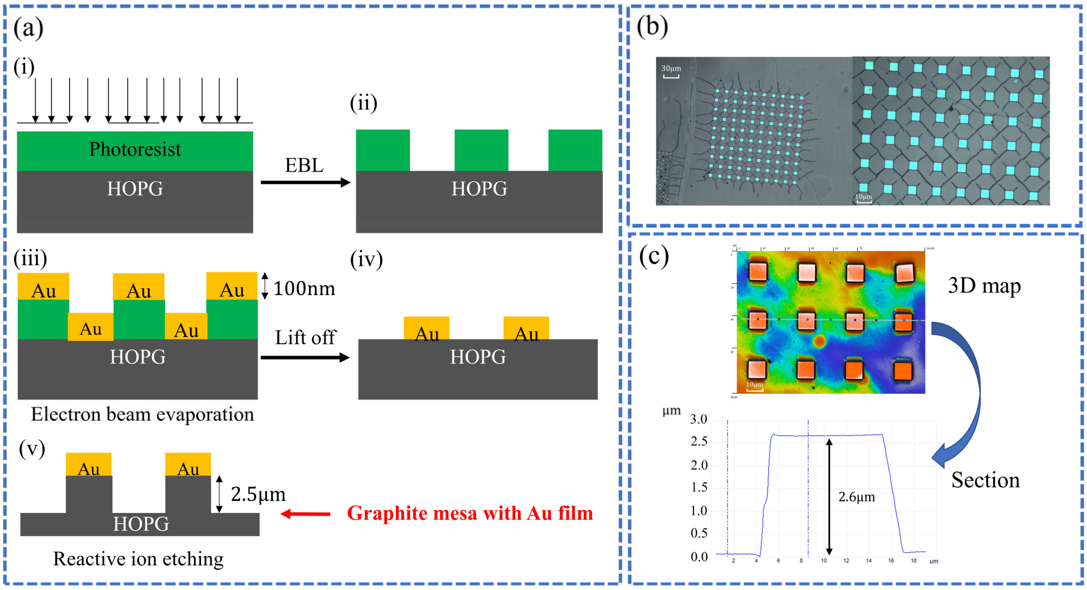

Supplementary Fig. 1 Fabrication process or graphite mesa with Au film and its characterizations: (a) Fabrication flow. (b) Optical image of fabricated graphite mesa with Au film. (c) Three-dimensional white light interference profile characterization of graphite mesa with Au film.

For the fabrication of n-Si, we used electron beam evaporation to deposit a layer of 100nm Al on one side of a 4-inch, 200um thick, double-polished silicon wafer with a <100> crystal plane, then used the wafer scriber to cut into small pieces of $1\text{cm} \times 1\text{cm}$ size, soaked the each piece in BOE (Buffered oxide etch) solution for 15 minutes to remove the oxide layer on the surface of n-Si, and finally cleaned with acetone, alcohol, and deionized water, encapsulate with vacuum.

2. The preparation of S-SLG

2.1 The transfer process of graphite flake

In order to form the S-SLG structure shown in Fig. 1a, we transferred graphite flake with single crystal superlubric interface to the n-Si surface, and the specific process is shown in Supplementary Fig. 2, where the left side of each figure is schematic diagram, and the right side is optical microscope observation. Firstly, we used a tungsten microtip controlled by a micromanipulator (Kleindiek MM3A) to attach the Au film of graphite mesa fabricated in Supplementary Section 0, as shown in Supplementary Fig. 2a, and applied a shear stress by the micromanipulator until they split a short distance from their vertical direction, as shown in Supplementary Fig. 2b. Secondly, we removed the microtip to observe whether the sheared graphite flake undergoes self-recovery motion (SRM) [2] to determine whether it has a single crystal superlubric interface [3], as shown in Supplementary Fig. 2c. Lastly, we re-attached the graphite flake which has SRM property with a microtip and split it out completely, as shown in Supplementary Figs. 2d and e, after that, we removed the dangling graphite flake dragged by the microtip, and placed it slowly by micromanipulator on the atomically smooth fabricated n-Si surface in Section 0, at this time, since the adsorption force of the graphite flake and n-Si is larger than that of the microtip and graphite flake, the graphite flake will remain

on the n-Si surface, as shown in Supplementary Fig. 2f, which formed the S-SLG structure shown in Fig. 1a. Since the interface of the finally transferred graphite flake was not exposed during the whole fabrication process in Supplementary Section 0, it would not be contaminated.

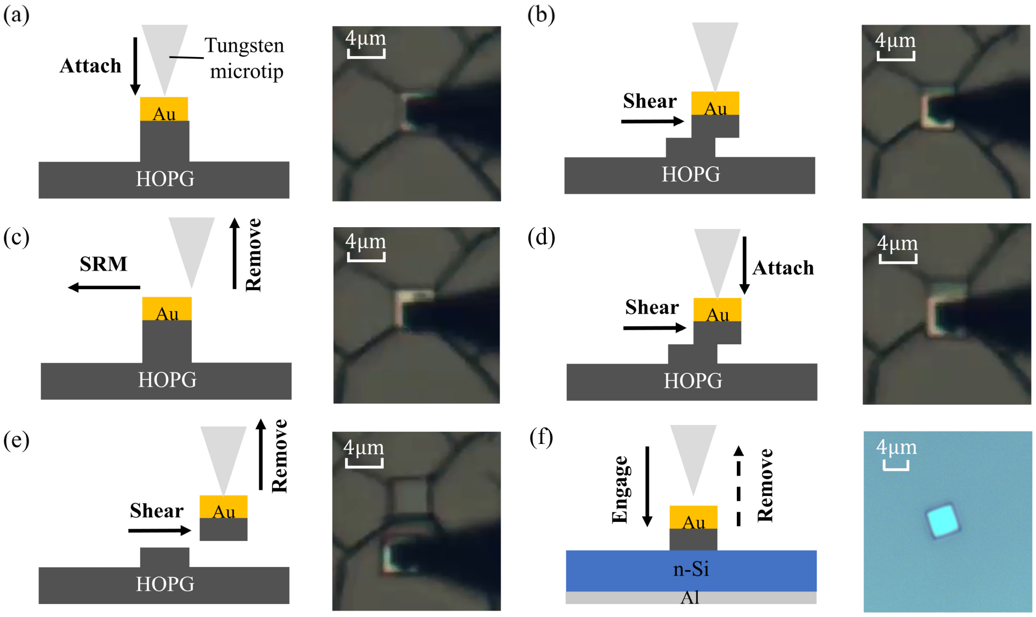

Supplementary Fig. 2 Preparation of graphite flake. (a) Attachment of microtip and graphite mesa with Au film. (b), (c) Observation of SRM. (d)-(f) Transfer process of graphite flake with SRM property.

2.2 The work function measurement of n-Si and HOPG

The work function of n-Si and HOPG was measured with the AFM tip coated Au (ACCESS-NC-GG(Appnano)) through Asylum Research Cypher S AFM in Scanning Kelvin Probe Microscope (SKPM) model. The material parameters of measured n-Si are consistent with Fig. 1a, and the HOPG surface is obtained by shearing cleavage. The results of the surface potential of n-Si ($V_{\mathrm{SP}}^{(\mathrm{Si})} = 0.682 - 0.739\ \mathrm{V}$) and HOPG ($V_{\mathrm{SP}}^{(\mathrm{gr})} = 0.275 - 0.331\ \mathrm{V}$) are shown in Supplementary Figs. 3a and b respectively by scanning a $2\ \mu\mathrm{m} \times 2\ \mu\mathrm{m}$ smooth area. According to the principle of SKPM[4], we can calculate the work function of n-Si ($W_{\mathrm{n-Si}}$) and HOPG (W_{gr}) as

$$W_{\mathrm{n-Si}} = W_{\mathrm{Au}} - eV_{\mathrm{SP}}^{(\mathrm{Si})} = 4.36 - 4.42\ \mathrm{eV},$$
$$W_{\mathrm{gr}} = W_{\mathrm{Au}} - eV_{\mathrm{SP}}^{(\mathrm{gr})} = 4.77 - 4.82\ \mathrm{eV}, \tag{1}$$

where $W_{\mathrm{Au}} = 5.1\ \mathrm{eV}$ is the work function of Au. According to the statistical theory of semiconductors[5] and known doping concentration $N_{\mathrm{D}} = 10^{15}\mathrm{cm}^{-3}$ of n-Si, the theoretical work function can be calculated by formula

$$W_{\mathrm{n-Si}}^{(\mathrm{theory})} = \chi_{\mathrm{n-Si}} - k_{\mathrm{B}}T\ln\left(\frac{N_{\mathrm{D}}}{N_{\mathrm{c}}}\right) = 4.29\ \mathrm{eV}, \tag{2}$$

where $\chi_{n-Si} = 4.05\ \mathrm{eV}$ is electron affinity of the n-Si, $N_{\mathrm{c}} = 2\left(\frac{2\pi m_{\mathrm{e}}^{*}k_{\mathrm{B}}T}{h^2}\right)^{\frac{3}{2}} = 2.5 \times 10^{19}\mathrm{cm}^{-3}$ is thermally excited state density of electron, $m_{\mathrm{e}}^{*} = 1.08m_0$ is effective electron mass, m_0 is the inertial mass of electrons, h is the Planck constant, k_{B} is the Boltzmann constant, and $T = 298.15\ \mathrm{K}$ is the temperature. The ideal theoretical calculation value is slightly lower than the measured value ($W_{\mathrm{n-Si}}^{(\mathrm{theory})} < W_{\mathrm{n-Si}}$), which might be caused by the surface state of silicon.

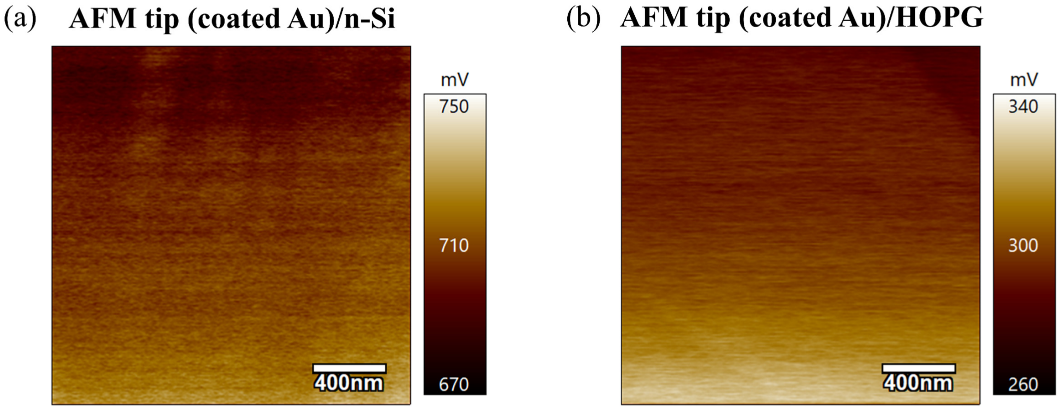

Supplementary Fig. 3 SKPM measurement results of n-Si surface and HOPG: (a) Surface potential between AFM tip coated Au and n-Si with doping concentration of $N_{\mathrm{D}} = 10^{15}\ \mathrm{cm}^{-3}$. (b) Surface potential between AFM tip coated Au and HOPG.

Furthermore, the work function of Al is $W_{\mathrm{Al}} = 4.28\ \mathrm{eV}$. Therefore, the ohmic contact ($W_{\mathrm{Al}} < W_{\mathrm{n-Si}}$) will be formed between the bottom Al electrode and n-Si of S-SLG.

3. Friction and current measurements of AFM system

The friction and current measurements of the graphite/n-Si heterostructures were performed under an ambient atmosphere. The experimental set-up included a commercial NTEGRA upright AFM (NT-MDT), a 100 μm XYZ piezoelectric displacement platform, a high numerical aperture objective lens (×100 (Mitutoyu)) and visualized conductive AFM tip (ACCESS-NC-GG(Appnano)). Fig. 1a shows the schematic of the experimental set-up. We accurately pressed the AFM tip on the Au cap of the graphite flake through the optical microscope and piezoelectric displacement platform. The AFM tip was calibrated in situ by the Sader method [6,7] for the normal direction force and the diamagnetic levitation spring system [8] for the lateral direction force. The bottom Al film of the n-Si is grounded through the iron stage, and the conductive AFM tip is also grounded by connecting a precision ammeter, which can accurately measure the current through AFM tip in the sliding process.

3.1 Calibration of AFM tip

Take the friction measurement in Fig. 1d as an example, the normal force F_{N} was applied by using the AFM cantilever, which can be written as

$$F_{\mathrm{N}} = \frac{k_{\mathrm{n}}}{S_{\mathrm{n}}} \times I_{\mathrm{n}}, \tag{3}$$

where S_{n} is the optical lever sensitivity, k_{n} is the normal spring constant, and I_{n} is optical detector signal. The lever sensitivity is measured by performing a standard force curve measurement as shown in Supplementary Fig. 4a, which includes loading (Forward, blue line) and unloading (Backward, red line) process. We also obtained average slope $S_{\mathrm{n}}^{(\mathrm{av})} = 0.0133\ \mathrm{nA/nm}$ through linear fitting. The normal spring constant $k_{\mathrm{n}} = 29.77\ \mathrm{N/m}$ was calibrated by the Sader method [6]. The optical detector signal $I_{\mathrm{n}} = 10\ \mathrm{nA}$ was set by AFM instrument. Therefore, we calculated the normal force of $F_{\mathrm{N}} = 22.3\ \mu\mathrm{N}$.

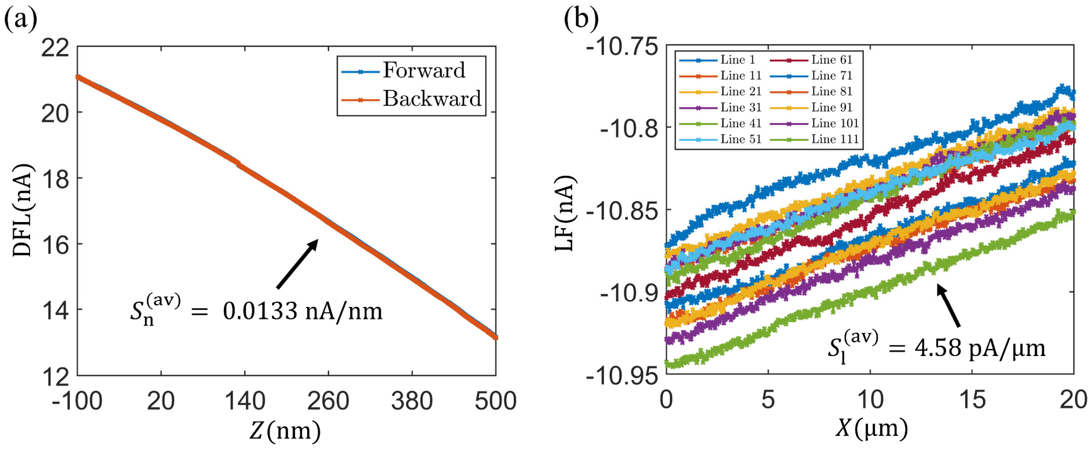

Supplementary Fig. 4 Lever sensitivity measurement for normal and lateral calibration: (a) Standard force curve measurement for normal calibration, which includes the forward (blue) and backward (red) process. (b) Diamagnetic lateral force curve measurement for lateral calibration, where different colours represent different test lines.

Similar to the normal force, the friction force f was measured by AFM cantilever, which can be written as

$$f = \frac{k_l}{S_l} \times I_l, \quad (4)$$

where S_l is the optical lever sensitivity, k_l is the lateral spring constant of diamagnetic levitation system[8], and I_l is measured frictional optical detector signal [8]. The $k_l = 3.05 \times 10^{-2}$ N/m was calibrated by using high-speed CCD to detect the vibration frequency of the levitate graphite sheet and precision balance to measure its mass[8]. The optical lever sensitivity was measured by using the AFM tip to drag the levitate graphite sheet and measure its force curve as shown in Supplementary Fig. 4b, we tested 122 times, and 12 of them are drawn in the figure, and we obtained its average slope $S_l^{(av)} = 4.58$ pA/μm through linear fitting. Therefore, we calculated the lateral force coefficient $\alpha = k_l/S_l = 6.66$ nN/pA.

3.2 Noise current measurement of AFM system

In order to detect the noise of AFM (NT-MDT) current measurement system, we adopted the same structure as in Fig. 1a, used the AFM tip static contact the graphite flake, and the measured noise current I_{noise} is shown in Supplementary Fig. 5a, where

the x-axis is 256 data points collected in each cycle (the frequency is 1Hz), and the y-axis corresponds to the time t of each cycle. We further averaged the 256 data points of each cycle to obtain the relationship between the average noise current $I_{\mathrm{noise,av}}$ and time t as shown in Supplementary Fig. 5b. It can be seen that the noise current is basically maintained at the order of 1pA, which is much smaller than the measured current in Fig. 1, and illustrates the reliability of the measured current.

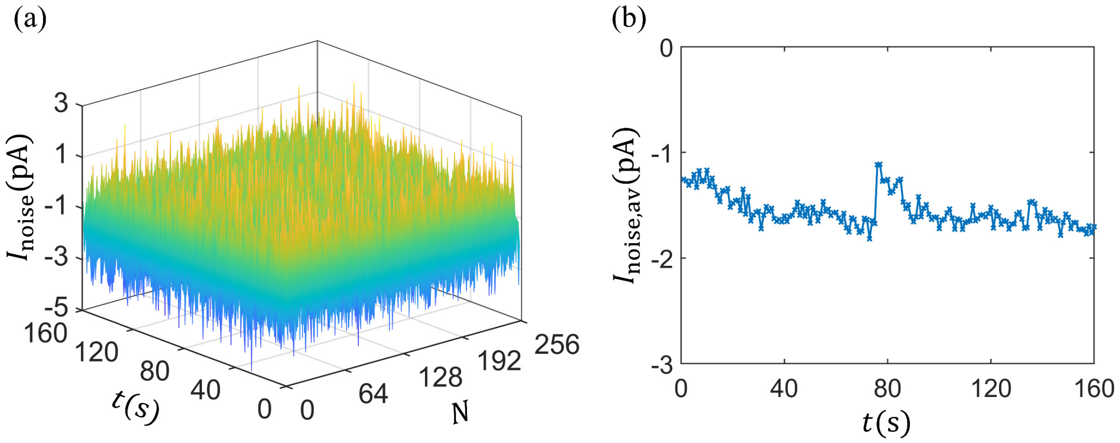

Supplementary Fig. 5 Noise current measurement for AFM (NT-MDT) system: (a) Noise current I_{noise} map for 160 cycles (the frequency is 1Hz), each cycle collects 256 data points. (b) Relationship between the average noise current $I_{\mathrm{noise,av}}$ and time t.

3.3 Current decay caused by graphite flake delamination

The graphite flake contains many incommensurate interfaces, which has probability of delamination during the sliding process, resulting in relative sliding between inner layers, and we analyzed its influence on S-SLG through an interesting experimental phenomenon. A total of four processes occurred:

(i) Overall sliding: The graphite flake was overall sliding relative to n-Si with the speed of $24\ \mu\mathrm{m/s}$ and normal force of $22.3\ \mu\mathrm{N}$, the optical microscope observation and schematic diagram are shown in Supplementary Fig. 6a, the measured current at this phase in Supplementary Fig. 6e is maintained at a high value of $I = 1.8\ \mathrm{nA}$, and the fricion force maintained at a low value of $f = 0.5\ \mu\mathrm{N}$.

(ii) Layered sliding: The delamination occured at the middle interface of graphite flake, the relative sliding occured between not only the layered interface, but also the bottom graphite flake and n-Si at the same time, the optical microscope observation and schematic diagram are shown in Supplementary Fig. 6b. The relative sliding between the layered interface was small when delamination just occured, and the relative sliding between the bottom graphite flake and n-Si was dominant, so the current in Supplementary Fig. 6e did not change much, but the measured friction increased slightly, which might be caused by the edges of layered interface. After that, the relative sliding of the bottom graphite/n-Si interface was reduced, so the measured current in Supplementary Fig. 6e was reduced to I = 0.2~0.7 nA.

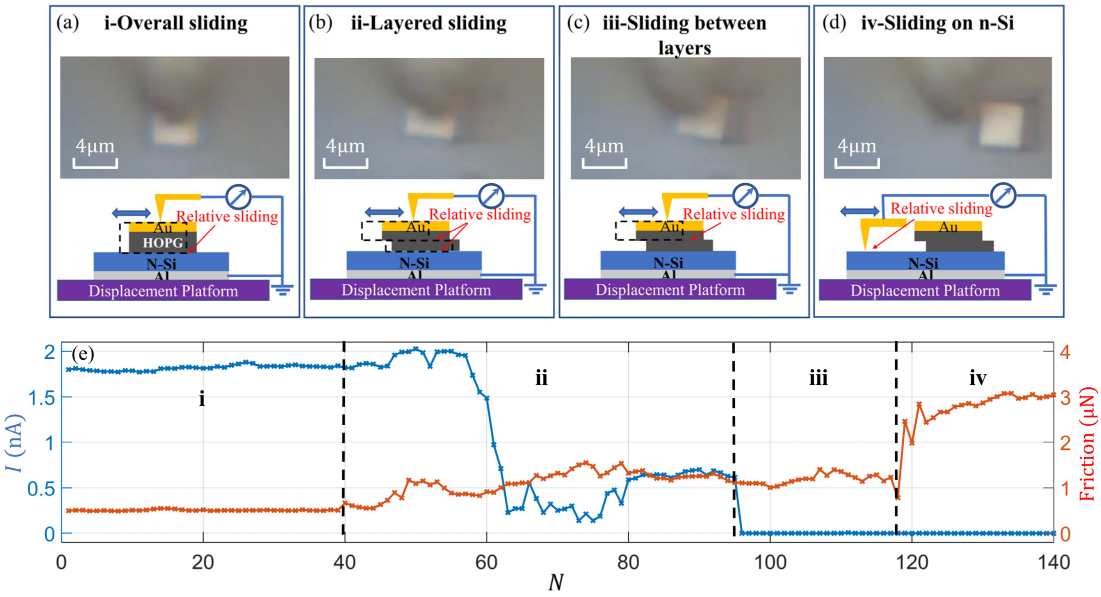

Supplementary Fig. 6 Output current and friction of four different relative sliding states: (a) Overall relative sliding state between graphite flake and n-Si. (b) Layerd relative sliding state, that is, there is not only relative sliding between the layered interface of the graphite flake, but also between the bottom graphite and n-Si. (c) Sliding between layers, that is, no relative sliding between bottom graphite and n-Si. (d) Relative sliding between AFM tip and n-Si (the upper is optical microscope observation and lower is schematic diagram). (e) Measured current I (blue) and friction (red) for the four different relative sliding state for (a)-(d), which use black dotted lines to distinguish, and the Arabic numerals i-iv represent the affiliation.

(iii) Sliding between layers: The relative sliding between the bottom graphite flake and n-Si stoped, and only occured between the layered interfaces, the optical microscope observation and schematic diagram are shown in Supplementary Fig. 6c. The measured current in Supplementary Fig. 6e droped to almost 0, while the friction force did not change significantly.

(iv) Sliding on n-Si: Finally, the AFM tip falled off from Au film on the top of the graphite flake and slided on the n-Si, the optical microscope observation and schematic diagram are shown in Supplementary Fig. 6d, while the measured current in Supplementary Fig. 6e was also close to 0 and the measured friction suddenly increased to ~3 μN, which indicates the low current and high friction stress characteristics of AFM tip/n-Si ordinary S-G.

The above experiments proove that the current generation of S-SLG is completely caused by the relative sliding at the graphite/n-Si interface rather than the relative sliding between the inner layer of the graphite flake.

4. Open-circuit voltage and power measurement

The open-circuit voltage and power measurements of the graphite/n-Si heterostructures were performed under an ambient atmosphere. The experimental set-up as shown in Supplementary Fig. 7a included a programmable micromanipulator (Kleindiek MM3A), a precision balance (Mettler) with resolution of 0.1 μN, a high numerical aperture objective lens (10×- 100× (Olympus)) and conductive tungsten probe with radius of 1 μm (prepared by electrochemical corrosion with 5 mol/L KOH solution). Supplementary Figs. 7b and c show the optical microscope image and actual photo of the experimental setup respectively. We accurately pressed the tungsten probe on the Au cap of the graphite flake through the optical microscope and micromanipulator, and accurately measured the applied normal force with the bottom precision balance. The bottom Al film of the n-Si was connected through an electrometer (KEITHLEY 6514) and the tungsten probe, which accurately measured the current and voltage in the sliding process.

4.1 Noise current and voltage measurement

In order to detect the noise current and voltage measurement of the above system, we adopted the same structure as Supplementary Fig. 7a and used the tungsten probe static contact the graphite flake with normal force $F_{\mathrm{N}} = 250\ \mu\mathrm{N}$. The measured noise current I_{noise} and noise voltage V_{noise} are shown in Supplementary Fig. 7d for 30 seconds, which were basically maintained at the order of $2\mathrm{pA}$ and $0.5\ \mathrm{mV}$ respectively. It is much smaller than the measured current and voltage in Fig. 2, and illustrates the reliability of the measured current and voltage.

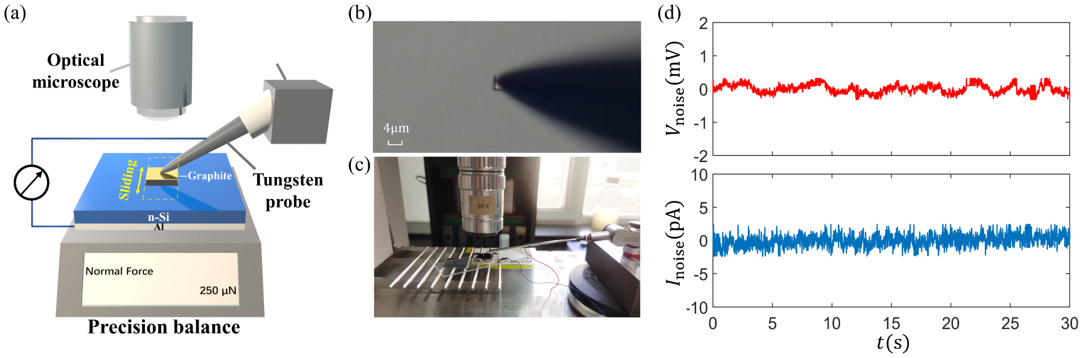

Supplementary Fig. 7 Measurement system for open-circuit voltage and power of S-SLG: (a) Structure of measurement system for graphite/n-Si S-SLG. (b) Optical microscope observation. (c) Actual photo of the experimental setup. (d) Noise current I_{noise} and voltage V_{noise} measurement of KEITHLEY 6514 with normal force of $F_{\mathrm{N}} = 250\ \mu\mathrm{N}$ as the function of time t.

4.2 The open-circuit voltage and short-circuit current waveform under different normal force

The tungsten probe was controlled by the micromanipulator to apply different normal force F_{N} which was detected by the bottom precision balance. The corresponding measured open-circuit voltage V_{oc} and short circuit current I_{sc} waveforms under some different F_{N} are shown in Supplementary Fig. 8, in which the sliding speed remained at $4.3\ \mu\mathrm{m/s}$. We statistically averaged the 4000 points in range

of $(70\% \times I_{sc}^{(max)} < I_{sc} < I_{sc}^{(max)}, 70\% \times V_{oc}^{(max)} < V_{oc} < V_{oc}^{(max)})$ for each waveform under different F_N to get Fig. 2b.

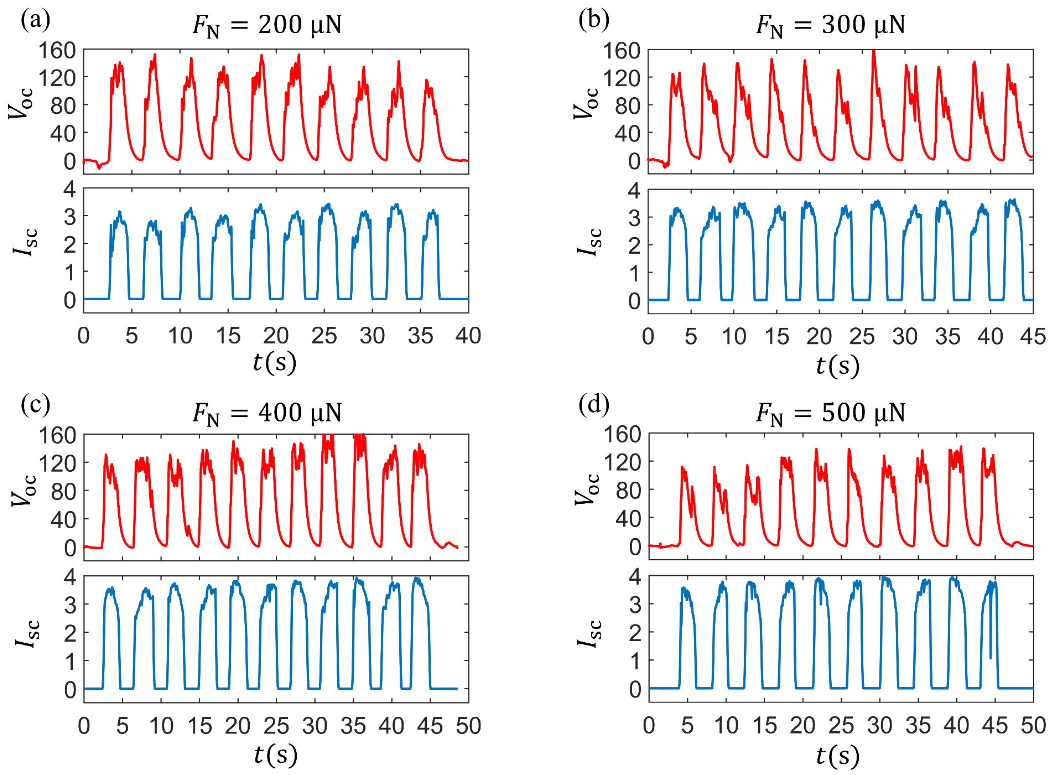

Supplementary Fig. 8 Open-circuit voltage V_{oc} (red) and short-circuit current I_{sc} (blue) waveform with time t under different normal force F_N with sliding speed of 4.3 μm/s: (a) $F_N = 200$ μN. (b) $F_N = 300$ μN. (c) $F_N = 400$ μN. (d) $F_N = 500$ μN.

4.3 The I-V characteristics of graphite/n-Si interface under different normal force

In order to verify the Schottky contact formed by the graphite flake and n-Si and explain the weak normal force correlation in Fig. 2b, we used the same system as shown in Supplementary Fig. 7a, replaced the electrometer with the KEITHLEY 2400 digital source meter, and measured the current-voltage (I-V) curves under different normal forrce as shown in Supplementary Fig. 9a (the positive direction of the bias voltage V_B is applied to the graphite flake relative to n-Si, and the sacn speed is 0.096 V/s), the

one-way conduction characteristic shows that a Schottky junction is formed between the graphite flake and n-Si ($W_{\mathrm{gr}} > W_{\mathrm{n-Si}}$), in which the forward conductivity is better for larger normal force. Subsequently, we used the thermionic emission model

$$I_{\mathrm{B}} = AA^{*}T^{2}\exp\left(-\frac{\Phi_{\mathrm{B}}}{k_{\mathrm{B}}T}\right)\exp\left(\frac{e(V_{\mathrm{B}}-I_{\mathrm{B}}R_{\mathrm{A}})}{nk_{\mathrm{B}}T}\right)\left(1-\exp\left(-\frac{e(V_{\mathrm{B}}-I_{\mathrm{B}}R_{\mathrm{A}})}{k_{\mathrm{B}}T}\right)\right), \quad (5)$$

of the system by considering the additional resistance R_{A} to fit the ideal factor n and Shottky barrier height Φ_{B} of data points, where $A^{*} = 252\ \mathrm{A/cm^{2}K^{2}}$ is effective Richardson constant, $A = 16\ \mu\mathrm{m}^{2}$ is the contact area, e is the electron charge, k_{B} is Boltzmann constant, $T = 283.15\ \mathrm{K}$ is the temperature.

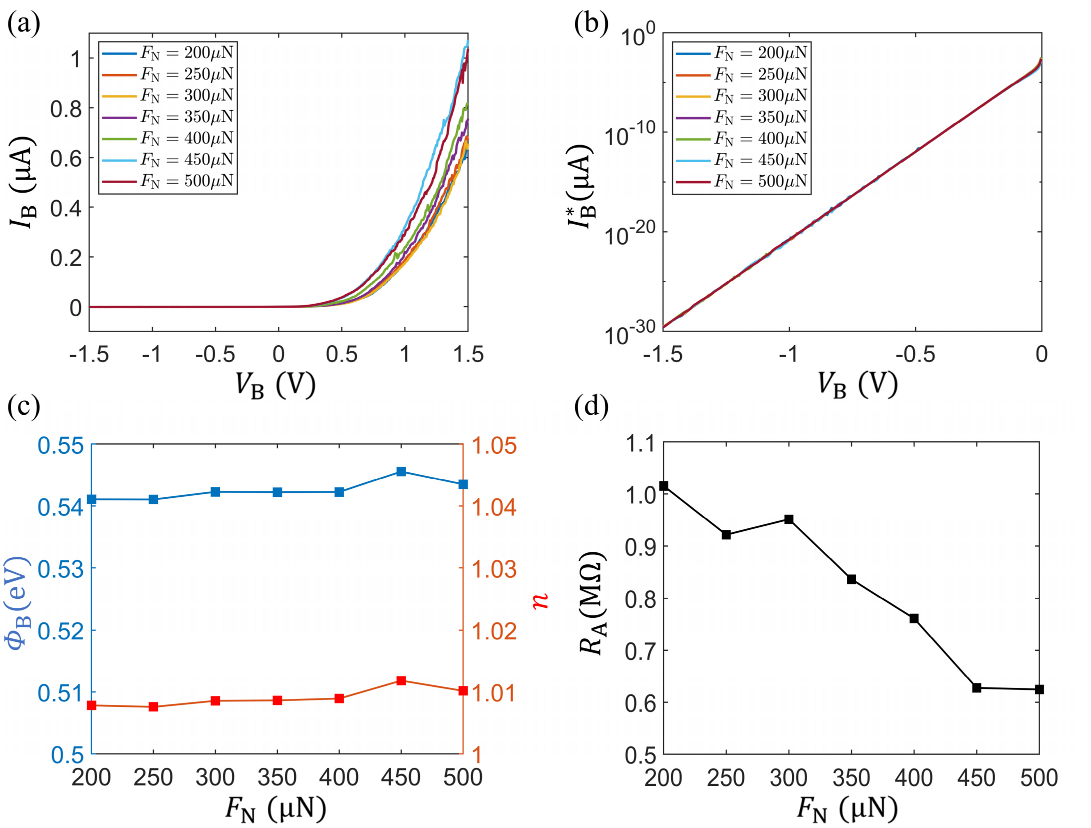

Supplementary Fig. 9 I-V characteristics of graphite/n-Si interface under different normal force. (a)Measured I-V curves under different normal force F_{N} (the abscissa is the bias voltage V_{B}, and the ordinate is the measured current I_{B}). (b) Relationship between effective current $I_{\mathrm{B}}^{*} = I_{\mathrm{B}}\left(1-\exp\left(-\frac{eV_{\mathrm{B}}}{k_{\mathrm{B}}T}\right)\right)^{-1}$ and V_{B} when $V_{\mathrm{B}} < 0$ under different normal force F_{N}. (c) Fitted ideal

factor n (red) and Schottky barrier height Φ_{B} (blue) according to Eq. (6) as function of normal force F_{N}. (d) Fitted additional resistance R_{A} according to Eq. (7) as function of normal force F_{N}.

We first focus on the inverse part ($V_{\mathrm{B}} < 0$), the current $|I_{\mathrm{B}}|$ is very small, that is, $|I_{\mathrm{B}}R_{\mathrm{S}}| \ll |V_{\mathrm{B}}|$, Eq. (5) can be simplified to

$$I_{\mathrm{B}}^{*} = AA^{*}T^{2}\exp\left(-\frac{\Phi_{\mathrm{B}}}{k_{\mathrm{B}}T}\right)\exp\left(\frac{eV_{\mathrm{B}}}{nk_{\mathrm{B}}T}\right), \quad (6)$$

where $I_{\mathrm{B}}^{*} = I_{\mathrm{B}}\left(1-\exp\left(-\frac{eV_{\mathrm{B}}}{k_{\mathrm{B}}T}\right)\right)^{-1}$ is effective current. The relationship between I_{B}^{*} and V_{B} is shown in Supplementary Fig. 9b, which showed exponential relationship (the linear relationship is shown in semi-logarithmic graph). The fitted ideal factor n and Schottky barrier height Φ_{B} under different normal force by Eq. (6) are shown in Supplementary Fig. 9c, where the $\Phi_{\mathrm{B}} \approx 0.54\ \mathrm{eV}$ and $n \approx 1.01$ showed normal force independence.

On the contrary, when $V_{\mathrm{B}} > 1V \gg k_{\mathrm{B}}T/e$, the Eq. (5) can be simplified to

$$V_{\mathrm{B}} = \frac{nk_{\mathrm{B}}T}{e}\log\left(\frac{I_{B}}{AA^{*}T^{2}}exp\left(\frac{\Phi_{B}}{k_{B}T}\right)\right) + I_{\mathrm{B}}R_{\mathrm{A}}. \quad (7)$$

Substituted the fitted n and Φ_{B} obtained in Supplementary Fig. 9c into Eq.(7) and used the data point of $V_{\mathrm{B}} > 1V$ to fit the additional resistance R_{A} under different normal forces as shown in Supplementary Fig. 9d, which shows a decreasing trend as the normal force increases. This trend explained the normal force correlation of short-circuit current, and the normal force independence of open-circuit voltage in Fig. 2b.

4.4 The open-circuit voltage measured by null method

As a double check, we further used the null method[9] through changing the bias voltage until the current disappeared to measure the open-circuit voltage of the graphite/n-Si S-SLG. We still used the same AFM system in Fig. 1a to measure output current while applying a bias voltage V_{b} to the sample, as shown in Supplementary Fig. 10a. Firstly, we measured the output current with speed of $4\ \mu\mathrm{m/s}$ by applying a sudden changed bias voltage in the order of $[150, -150, 150, -150]\ \mathrm{mV}$ as shown in

Supplementary Fig. 10b, and each bias voltage was measured around 64 cycles, with the total of 256 cycles. When $V_{\mathrm{b}} = 150$ mV, the output current was significant. The maximum current was up to about 2.5nA, and when $V_{\mathrm{b}} = -150$ mV, the output current disappeared immediately. It shows that the direction of the output voltage of the external circuit is from graphite flake to n-Si, which is consistent with the direction measured in Fig. 2.

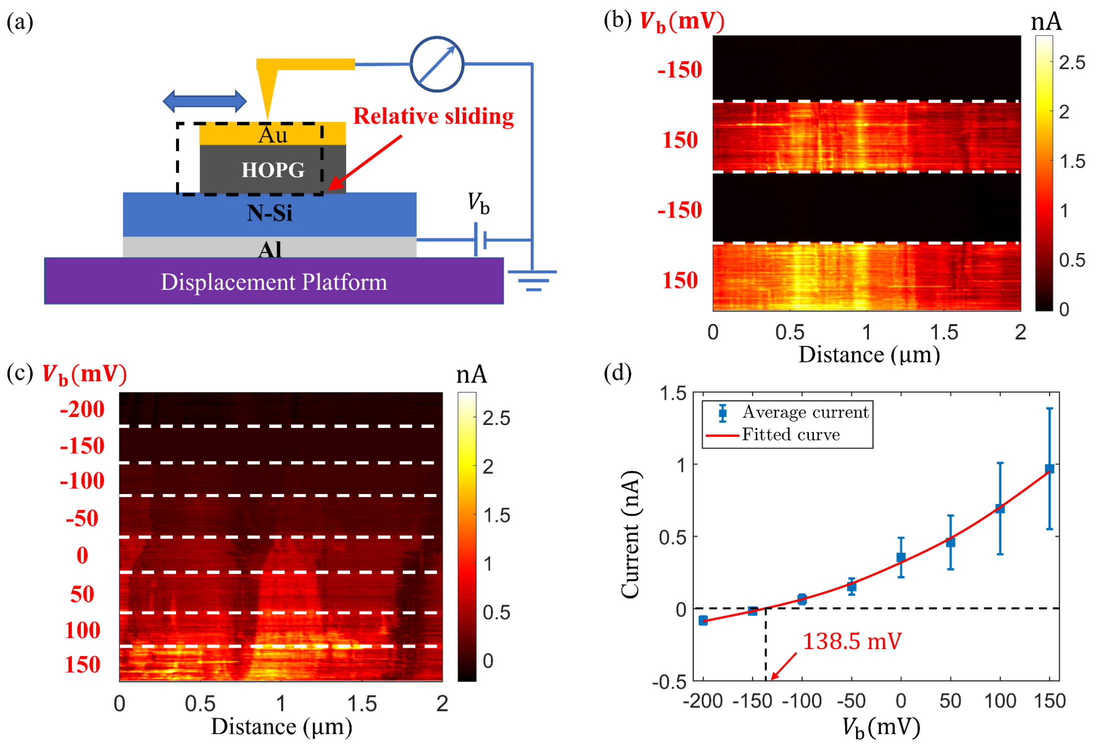

Supplementary Fig. 10 Null method measurement of the open-circuit voltage of Graphite/n-Si S-SLG: (a) Experimental set-up. (b) Current maps for 256 cycles with speed of $4\ \mu\mathrm{m/s}$ under bias voltage $V_{\mathrm{b}} = 150$ mV or -150 mV. (c) Current maps for 256 cycles with speed of $4\ \mu\mathrm{m/s}$ under gradual bias voltage V_{b} from 150 mV to -200 mV. (d) Average output current as the function of bias voltage V_{b} (blue scatter points, each point and error bar were obtained by the data corresponding to different V_{b} in (c)), the red line is fitted by cubic spline, which the open-circuit voltage corresponding to the output current equal to zero is 138.5 mV.

We gradually changed the bias voltage V_{b} from 150 mV to -200 mV at intervals of 50 mV to obtain the output current as shown in Supplementary Fig. 10c. We obtained the average current under different bias voltages as shown in Supplementary

Fig. 10d (blue scatter points), and fit by cubic spline (red line). We obtained the open-circuit voltage corresponding to the output current equal to zero is $V_{\mathrm{oc}}^{(\mathrm{null})} = 138.5\ \mathrm{mV}$, which is consistent to the result in Fig. 2 and confirms the accuracy of the measurement.

5. The contact area calculation of AFM tip/n-Si ordinary S-G

For AFM tip/n-Si ordinary S-G, the DMT model [10] can best approximate the contact between the AFM tip and the hard poorly adhesive material. According to the DMT model [10], the contact area A is given by:

$$A = \pi\left(\frac{R}{K}(F_{\mathrm{N}} + 2\pi R\gamma)\right)^{\frac{2}{3}}, \tag{8}$$

where R is AFM tip radius, F_{N} is the normal force applied to the AFM tip, γ is the energy of adhesion, the term $2\pi R\gamma$ can be considered as an additional load, which is determined by the "pull-off" force in the force curve, and K is the reduced Young's modulus given as

$$K^{-1} = \frac{3}{4}\left(\frac{1-\nu_{\mathrm{s}}^2}{E_{\mathrm{s}}} + \frac{1-\nu_{\mathrm{t}}^2}{E_{\mathrm{t}}}\right) \tag{9}$$

where E_{t} and E_{s} are Young's modulus of the tip and the sample, and ν_{t} and ν_{s} are the Poisson ratios of tip and sample, respectively. For the AFM tip/n-Si ordinary S-G shown in Fig. 1e, the parameters of AFM tip (made of silicon and coated with Au, the model is HA_NC/Au (NT-MDT & TipsNano)) are $E_{\mathrm{t}} = 190\ \mathrm{GPa}$, $\nu_{\mathrm{t}} = 0.28$ and $R = 35\ \mathrm{nm}$, the parameters of n-Si surface are $E_{\mathrm{s}} = 190\ \mathrm{GPa}$, $\nu_{\mathrm{s}} = 0.28$, from Eq. (9) we obtained $K = 137.44\ \mathrm{GPa}$. Through the same calibration method as in Supplementary Section 3.1, we obtained the normal force applied to AFM tip which is $F_{\mathrm{N}} = 4.49\ \mu\mathrm{N}$, and in order to obtain the "pull-off" force, we performed a force curve measurement as shown in Supplementary Fig. 11, there is a negative valley (the black dashed box area) of retraction process, the illustration is the partial zoom, which the valley value is the adhesion "pull-off" force $2\pi R\gamma = 65.9\ \mathrm{nN}$, therefore, we calculated $A = 343.52\ \mathrm{nm}^2$ through Eq. (8).

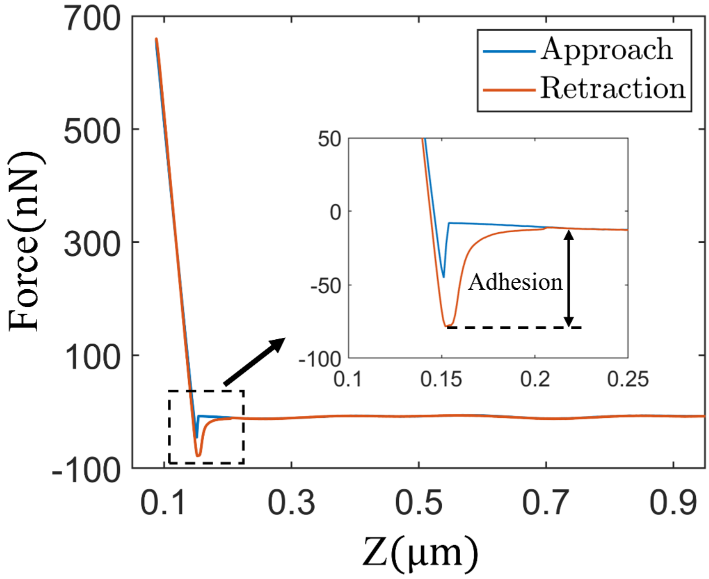

Supplementary Fig. 11 Force curve measurement of AFM tip/n-Si ordinary S-G, where the vertical axis is the height of the pressed probe Z.

Through the above analysis, we conclude that the "pull-off" force is much smaller than the normal force applied to AFM tip ($2\pi R\gamma \ll F_{\mathrm{N}}$), which can be ignored in Eq. (8) and the Hertz contact model [11,12] could replace DMT contact model [10] to consider the normal pressure distribution

$$P_{\mathrm{N}} = P_0\left(1 - \frac{r^2}{a^2}\right)^{\frac{1}{2}}, \tag{10}$$

of the contact between AFM tip and n-Si, where $r \leq a$ is the distance from the center in polar coordinates, $a = \left(\frac{F_{\mathrm{N}}R}{K}\right)^{\frac{1}{3}}$ is the contact radius ($A = \pi a^2$), $P_{\mathrm{av}} = \frac{F_{\mathrm{N}}}{A}$ is the average normal pressure, and $P_0 = \frac{3}{2}P_{\mathrm{av}}$ is the maximum normal pressure at the position of contact center ($r = 0$). For friction shear stress P_{s}, we assume that the friction coefficient μ of the whole contact region are the same ($P_{\mathrm{s}} = \mu P_{\mathrm{N}}$), which means P_{s} has the same distribution

$$P_{\mathrm{s}} = P_{\mathrm{f}}\left(1 - \frac{r^2}{a^2}\right)^{\frac{1}{2}}, \tag{11}$$

as P_{N}, where

$$P_{\mathrm{f}} = \frac{3}{2}\frac{f}{A}, \tag{12}$$

is the maximum friction shear stress of the contact region, and f is the measured friction force.

6. Verification of the SSL state in graphite/n-Si S-SLG

6.1 Surface characterization method of tribological experiment

In order to verify the contact between the graphite flakes and n-Si in the S-SLG is in the state of structural superlubricity (SSL), we conducted a series of tribological tests as shown in Fig .3. Here, we show the specific process and method of the experiment.

The first step is characterized the two sliding interfaces, for graphite flake surface, we used the method described in Supplementary Section 2.1 to select graphite flake with SRM [2] property (Supplementary Figs. 2a-c), and used Asylum Research Cypher S AFM in tapping mode to characterize the topography of interface (Fig. 3a) through flipped 180 degree of microtip after adsorbing the graphite flake (Supplementary Fig. 2e) before placing it on the n-Si surface (Supplementary Fig. 2f), and characterized the topography of n-Si surface (Fig. 3b) at the same time.

For the second step, we used the lateral force measurement system of AFM (NT-MDT) to measure the relationship between friction force and normal force with displacement amplitude of $2\ \mu m$ and speed of $4\ \mu m/s$, where each point was tested 40 times. Then we fitted the corresponding friction coefficient (Fig. 3c), and measured the friction force of 6,000 cycles sliding process with displacement amplitude $4 \mu m$ and speed $8 \mu m/s$ under a normal force of $F_N = 23.8\ \mu N$ (Fig. 3d).

For the third step, we performed some characterizations on the two interfaces after 6,000 sliding cycles. For the slided n-Si surface, we firstly used Asylum Research Cypher S AFM in tapping mode to perform a larger range morphological characterization and found the position of the graphite flake as shown in Supplementary Fig. 12, that is, located the sliding region (the yellow dashed frame), and further characterized the small sliding region of n-Si through the positioning function of AFM, to judge whether there is any observable damage on slided n-Si surface (Fig. 3e).

Large region characterization

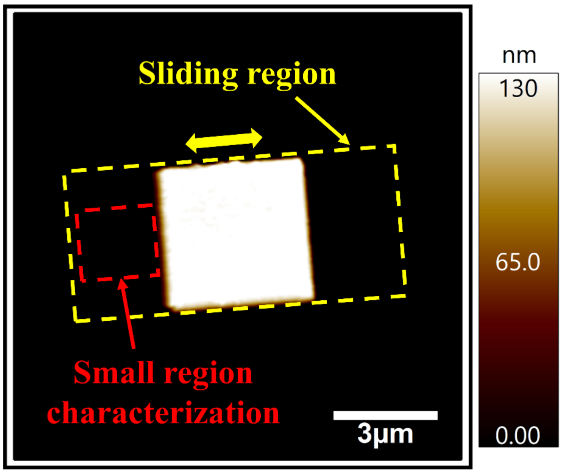

Supplementary Fig. 12 Larger range morphological characterization of the graphite flake and slided n-Si surface, where the yellow dashed frame is the sliding region, and the red dashed frame is the small region for next characterization step.

For the fourth step, we performed a Raman characterization (LabRAM HR Evolution Raman spectrometer from HORIBA, with resolution of $0.1\ \mathrm{cm}^{-1}$, laser wavelength of $532\ \mathrm{nm}$, grating of 1800 (450-850 nm), acq. time of 4s and spot diameter of $1\ \mu\mathrm{m}$) on the slided n-Si interface. The intensity distribution of the *G* peak ($1580\ \ \mathrm{cm}^{-1}$) in the range of $20\ \mu\mathrm{m} \times 20\ \mu\mathrm{m}$ was obtained by using the mapping scan mode as shown in Supplementary Fig. 13a. The area with higher intensity in the middle is the position of the graphite flake (the laser penetrates the Au film detect the graphite underneath). No obvious change in the intensity of the *G* peak on slided n-Si region was around the graphite flake, and all were at the reference value. Further, the Raman characterization results at different positions (points 1-6) on the slided n-Si interface are shown in Supplementary Fig. 13b. No observable *G* peak ($1580\ \ \mathrm{cm}^{-1}$) indicates that there was no visible graphite wear debris on the slided n-Si interface after 6,000 sliding cycles.

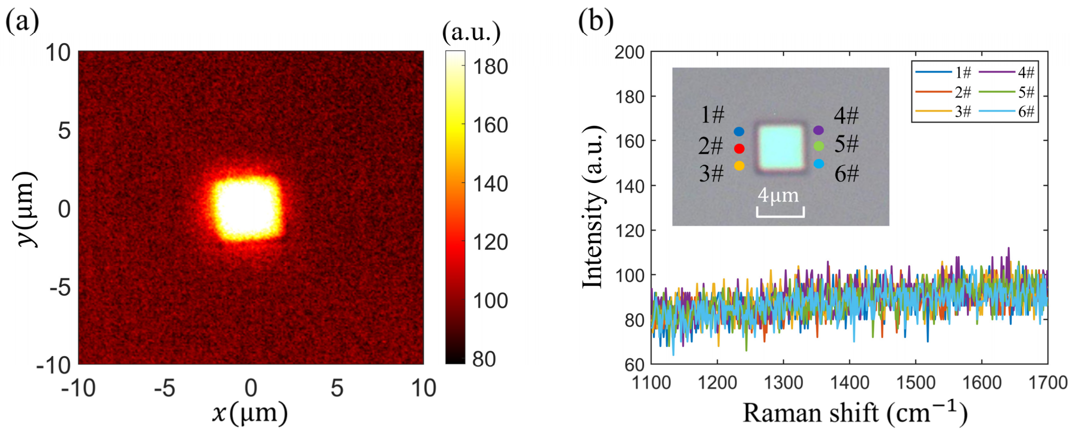

Supplementary Fig. 13 Raman characterization of slided n-Si interface: (a) Intensity distribution of the *G* peak (1580 cm^{-1}) in the range of $20\ \mu m \times 20\ \mu m$. (b) Raman characterization results at different positions (points 1-6, illustration is the position of each point) on the slided n-Si interface.

Before characterizing the slided graphite flake interface, we firstly used the method shown in Supplementary Fig. 14 to lift the graphite flake by overcoming the van der Waals adsorption force between graphite flake and n-Si interfaces. The whole process consists of three steps:

- Step 1: We placed the AB glue mixed in a ratio of 1:1 on the glass slide, and put it in the ambient atmosphere for 30 mins, and then we used a tungsten microtip controlled by a micromanipulator (Kleindiek MM3A) to extend into the AB glue (Supplementary Fig. 14a). There would be a small glue drop (the diameter is around 4 μm) attached to the microtip (Supplementary Fig. 14b) when we pulled out the microtip. The Supplementary Fig. 14c is the microscope observation after pulling out the microtip, and the yellow dashed frame is the position of the small glue drop.
- Step 2: We aligned the microtip with small glue to the top of slided graphite flake (Supplementary Fig. 14d), and then used the micromanipulator to stick the small glue drop with the graphite flake (Supplementary Fig. 14e). The Supplementary Fig. 14f is the microscope observation of the sticking process.
- Step 3: When the small glue drop is solidified and bonding, the bonding force between the small glue drop and the graphite flake will be larger than the van der

Waals adsorption force between the graphite flake and n-Si, so we could lift the graphite flake by the micromanipulator (Supplementary Fig. 14g).

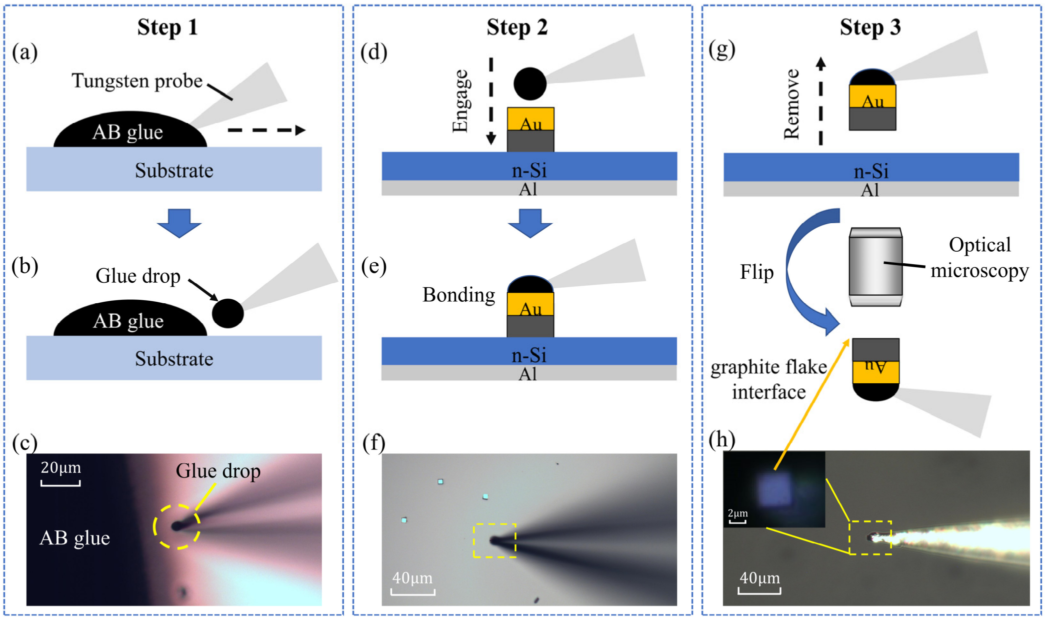

Supplementary Fig. 14 Method to stick and flip graphite flake by use small glue drop: Step 1: (a) Use a tungsten microtip extend into the AB glue. (b) Pull out the microtip attach a small glue drop (the diameter is around 4 μm). (c) Microscope observation after pulling out process. Step 2: (d) Alignment process of microtip with small glue drop and graphite flake. (e) Sticking process of graphite flake and small glue drop. (f) Microscope observation of sticking process. Step 3: (g) Lifting and flipping process of the graphite flake from n-Si surface. (h) Microscope observation of slided interface of graphite flake.

After the above process, we flipped 180 degree of microtip to observe the slided interface of graphite flake by optical microscope as shown in Supplementary Fig. 14h, where the illustration in the upper left corner is a partial enlarged view (the region within the yellow dashed frame). Furthermore, we performed the Raman characterization on the flipped slided graphite flake interface (Fig. 3f), to judge whether there is any observable damage from whether there is a *D* peak (1350 cm^{-1}).

6.2 The graphite defects resolution estimation of Raman measurement

Due to the limitations of Raman equipment, the measured results have a certain degree of noise. Therefore, we cannot observe *D* peaks ($1350\ \mathrm{cm}^{-1}$) below the noise level. And the defect density ρ_{d} of graphite can be obtained according to the intensity ratio of *D* peak ($1350\ \mathrm{cm}^{-1}$) I_{D} and *G* peak ($1580\ \mathrm{cm}^{-1}$) I_{G}, which

$$\rho_{\mathrm{d}} = k\left(\frac{I_{\mathrm{D}}}{I_{\mathrm{G}}}\right)^2 \tag{13}$$

where $k = 0.022$. According to the Raman results in Fig. 3f, we can obtain that the maximum value of $I_{\mathrm{D}}/I_{\mathrm{G}}$ is around 0.0597, and the defect density ρ_{d} is around 7.84×10^{-5}, which is the graphite defects resolution of our Raman measurement. If the density of the defect is less than this low value, we cannot distinguish based on the currently Raman measurement.

7. The tunnelling probability of electron through Schottky barrier in S-SLG

According to the principle of tunneling effect[13], for a certain distributed potential barrier $U(x)$, the electron with lower energy E than the height of the barrier has a probability to tunnel the barrier, and the corresponding probability T_{t} is

$$T_{\mathrm{t}} = e^{-2\gamma}, \quad \gamma = \int_0^{L_{\mathrm{D}}} \frac{\sqrt{2m_{\mathrm{e}}^*(U(x) - E)}}{\hbar} dx\ , \tag{14}$$

Where $m_{\mathrm{e}}^* = 1.08m_0$ is effective electron mass, m_0 is the inertial mass of electrons, $\hbar$ is the Planck constant, $E = 0.0287\ \mathrm{eV}$ is the upper limit of friction energy generated by the interaction of each dangling bond with silicon atoms, which is calculated in **Discussion on the mechanism of S-SLG** Section in manuscript, and

$$L_{\mathrm{D}} = \sqrt{\frac{2\varepsilon(\mathrm{W_{gr}} - W_{\mathrm{n-Si}})}{e^2 N_{\mathrm{D}}}} = 728.2\ nm, \tag{15}$$

is the depletion layer width[5], where $\varepsilon = 11.7\,\varepsilon_0$ is the relative permittivity of silicon, ε_0 is the permittivity of vacuum, e is the electron charge, $W_{gr} = 4.80\,eV$ and $W_{n-Si} = 4.39$ eV are the average measured work function of graphite flake and n-S respectively in Supplementary Section 2.2, $N_D = 10^{15}$ cm^{-3} is the doping concentration of n-Si. Taking the contact interface as the origin, the potential distribution function can be written as

$$U(x) = \Phi_B - \frac{e^2 N_D}{2\varepsilon} x^2, \tag{16}$$

where $\Phi_B = 0.54$ eV is fitted by current–voltage (I–V) characteristic curve as shown in Supplementary Fig. 9c. Substituting Eq. (15) and Eq. (16) into Eq. (14), we can obtain $\gamma = 2334.4$, which indicates the probability of tunneling $T_t \approx 10^{-2028}$ is almost zero in our S-SLG for power generation process.

8. The quasi-static simulation of DLED mechanism

8.1 The setup of simulation

The geometric parameters and boundary conditions of the model were set as shown in Supplementary Fig. 15a. The model consists of a slider (length $L_1 = 4\mu m$ and height $h_1 = 1\mu m$) at the top and a much larger stator (length $L_2 = 20\mu m$ and height $h_2 = 10\mu m$) at the bottom. The stator is made of n-Si with a doping concentration of $N_D = 10^{15} cm^{-3}$. The slider is made of a heavily doped p$^+$-Si with a doping concentration of $N_A = 2 \times 10^{19}$ cm^{-3} as an equivalent metal. The interface between the stator and the slider (yellow solid line) was set to a continuous heterojunction condition

$$\begin{aligned} E_{fn}^{(1)} &= E_{fn}^{(2)}, \\ E_{fp}^{(1)} &= E_{fp}^{(2)}, \\ \overrightarrow{\boldsymbol{D_1}} &= \overrightarrow{\boldsymbol{D_2}}, \end{aligned} \tag{17}$$

where $E_{fn}^{(i)}$ and $E_{fp}^{(i)}$ ($i = 1,2$ represents the slider and stator) are the fermi levels of electrons and holes respectively. The top surface of the slider and the bottom surface of

slider were set to metal contact boundary, which are conductively connected through an external circuit series with a resistance R. The potential of the bottom surface of stator was set to be 0, and the potential of the top surface of the slider V_1 was determined according to the continuous conditions of the external current I_{R} through resistance

$$\begin{gathered} V_1 = I_{\mathrm{R}} R, \\ I_R = \iint I_{\mathrm{n}} dS, \end{gathered} \tag{18}$$

where I_{n} is the normal current on the bottom surface of the stator as shown in Supplementary Fig. 15a. The potential distribution V in the semiconductor satisfies the Poisson equation

$$\nabla \cdot (\varepsilon \nabla V) = q(n - p - N_D + N_A), \tag{19}$$

where ε is the permittivity, n and p are the electron and hole concentration respectively. The relationship between carrier concentration and energy band is given by statistical theory

$$\begin{gathered} n = N_{\mathrm{c}} \exp\left(-\frac{E_{\mathrm{c}} - E_{\mathrm{fn}}}{kT}\right), \\ p = N_{\mathrm{v}} \exp\left(\frac{E_{\mathrm{v}} - E_{\mathrm{fp}}}{k_{\mathrm{B}} T}\right), \\ E_{\mathrm{c}} = -\chi_{\mathrm{n-Si}} - qV, \\ E_{\mathrm{v}} = -\chi_{\mathrm{n-Si}} - E_{\mathrm{g}} - qV, \end{gathered} \tag{20}$$

where $N_{\mathrm{c}} = 2\left(\frac{2\pi m_{\mathrm{e}}^* k_{\mathrm{B}} T}{\hbar^2}\right)^{\frac{3}{2}}$ and $N_{\mathrm{v}} = 2\left(\frac{2\pi m_{\mathrm{h}}^* k_{\mathrm{B}} T}{\hbar^2}\right)^{\frac{3}{2}}$ are the thermally excited state density of electron and hole, E_{c} and E_{v} are the conduction band and valence band energy levels, $E_{\mathrm{g}} = 1.12\ \mathrm{eV}$ and $\chi_{\mathrm{n-Si}} = 4.05\ \mathrm{eV}$ are the band gap and electron affinity of n-Si respectively. By solving equations Eq. (19) and Eq. (20), we obtained the potential distribution and carrier concentration distribution in the semiconductor, and further, we calculated the electron and hole currents ($\overrightarrow{\boldsymbol{J}_{\mathbf{n}}}$ and $\overrightarrow{\boldsymbol{J}_{\mathbf{p}}}$) by the drift diffusion model

$$
\begin{aligned}
\overrightarrow{\boldsymbol{J}_{\mathbf{n}}} &= -qn\mu_{\mathrm{n}}\nabla V + qD_{\mathrm{n}}\nabla n, \\
\overrightarrow{\boldsymbol{J}_{\mathbf{p}}} &= -qp\mu_{\mathrm{p}}\nabla V - qD_{\mathrm{p}}\nabla p, \\
\nabla \cdot \overrightarrow{\boldsymbol{J}_{\mathbf{n}}} &= q\frac{\partial n}{\partial t}, \\
\nabla \cdot \overrightarrow{\boldsymbol{J}_{\mathbf{p}}} &= -q\frac{\partial p}{\partial t},
\end{aligned}
\tag{21}
$$

where μ_{n} and D_{n} are the electron mobility and diffusion coefficient respectively. The μ_{p} and D_{p} are the hole mobility and diffusion coefficient respectively.

8.2 The simulation process

The whole quasi-static simulation process was divided into three steps:

Step 1: Firstly, we simulated the electron and hole distribution when the Schottky diode was formed to reach static equilibrium ($\Delta x = 0$) by the slider and stator. The mesh division is shown in Supplementary Fig. 16a, and the simulation result of electron concentration distribution is shown in Supplementary Fig. 15b. It can be seen that a depletion layer is produced in n-Si. We further made a cut line in the middle ($x = 0$) to observe the distribution of energy band E, electric field in the y direction E_{y}, the logarithm of the electron concentration $\log(n)$ and hole concentration $\log(p)$ as shown in Supplementary Fig. 15c, where the orange dotted line is the location of the heterojunction interface, and the green region is the depletion layer, which penetrates primarily into the n-Si region, and the width of depletion in the p^{+}-Si region can be neglected. It indicates that the space charge region in p^{+}-Si is almost distributed at the interface, and the built-in electric field is also primarily distributing in n-Si region, which is similar to metal/semiconductor contact. Therefore, the above results illustrate the rationality of using heavily doped p^{+}-Si as an equivalent metal.

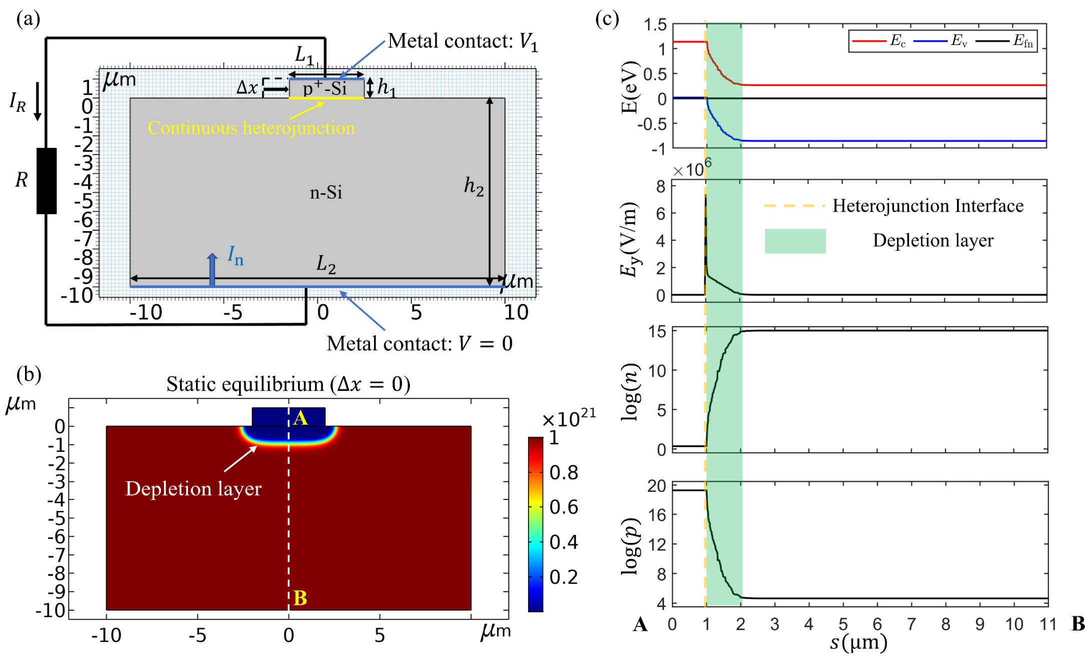

Supplementary Fig. 15 Structural sketch of quasi-static semiconductor finite element simulation with relevant parameters and its static equilibrium results: (a) Schematic diagram of the geometric parameters and boundary conditions. (b) Electron concentration distribution in static equilibrium ($\Delta x = 0$). (c) Variable distribution of AB cut line, from top to bottom are the energy band E, electric field in the y direction (upward is positive) E_y, the logarithm of the electron concentration $\log(n)$, and the logarithm of the hole concentration $\log(p)$, where the units of n and p are cm^{-3}.

Step 2: After equilibrium, we moved the upper slider with a displacement of $\Delta x = 0.5\mu m$ with a contrived constraint that the electron distributions of both the slider and the stator would not change, as shown in Fig. 4a, and the carrier distribution had reached a non-equilibrium state.

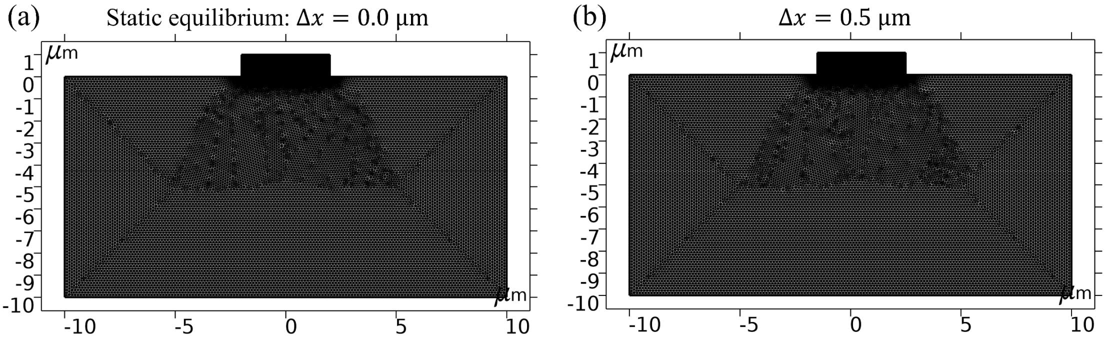

Supplementary Fig. 16 Meshing for quasi-static finite element simulation: (a) Meshing for static equilibrium solution ($\Delta x = 0$, Step 1). (b) Meshing for non-equilibrium instantaneous simulation ($\Delta x = 0.5$ μm, Step 3).

Step 3: We removed the constraint and started the transient simulation with the state of the step 2 as the initial condition, and the meshing is shown in Supplementary Fig. 16b, to simulate the transport process of carriers, electric potential distribution and related parameters (energy band distribution of cutline $\mathrm{A_1B_1}$ and $\mathrm{A_2B_2}$) over time.

8.3 The unbalance electric potential and electric field distribution

The simulated output electron current I_n along the bottom surface of stator with time, relationship between the electron concentration distribution with time, and the energy band distribution where the depletion layer establishment ($\mathrm{A_1B_1}$, $x = 2.6$ μm) and destruction ($\mathrm{A_2B_2}$, $x = -2.1$ μm) are shown in Figs. 4b-e, respectively. The above simulation indicates that the output current is mainly contributed by the electron drift motion caused by the non-equilibrium electric field during the movement of slider. In order to illustrate this conclusion, we showed the electric potential distribution at the time corresponding to Fig. 4c in Supplementary Fig. 17a, where the black arrow represents the direction of the electric field. The electric field in y-direction E_y at $\mathrm{A_1B_1}$ and $\mathrm{A_2B_2}$ in Supplementary Fig. 17a are shown in Supplementary Figs. 17b and c, respectively. It can be seen that when $t = 0$ ps, the E_y of $\mathrm{A_1B_1}$ and $\mathrm{A_2B_2}$ at the bottom surface of stator were all positive, which caused the electrons to drift and move out along the bottom surface of stator. Therefore, I_n at $t = 0$ ps was mainly contributed by the drift motion of the electron due to the unbalanced electric field, and the degree of the E_y decreases when $t = 1$ ps shown in Supplementary Figs. 17b and c, which explains the attenuation of I_n when $0 \text{ ps} < t < 2.3$ ps in Fig. 4b. When $t > 2.3$ ps, that is the E_y in $t = 3$ ps shown in Supplementary Figs. 17b and c, the E_y near the bottom surface of the stator approached to zero, and it has positive value

at the A_1B_1 and negative value at the A_2B_2 close to the depletion layer ($s < 2$ μm), that is, the electrons are mainly transferred inside to reach equilibrium rather than the external circuit. At last, when $t = 27$ ps, as shown in Supplementary Figs. 17b and c, the whole region reaches a new equilibrium state, which I_n decays to zero, and the E_y of A_1B_1 converges to the electric field of the static equilibrium depletion layer (the E_y is only appears in the depletion layer), and the E_y of A_2B_2 overall approach to 0 (the depletion layer destruct completely).

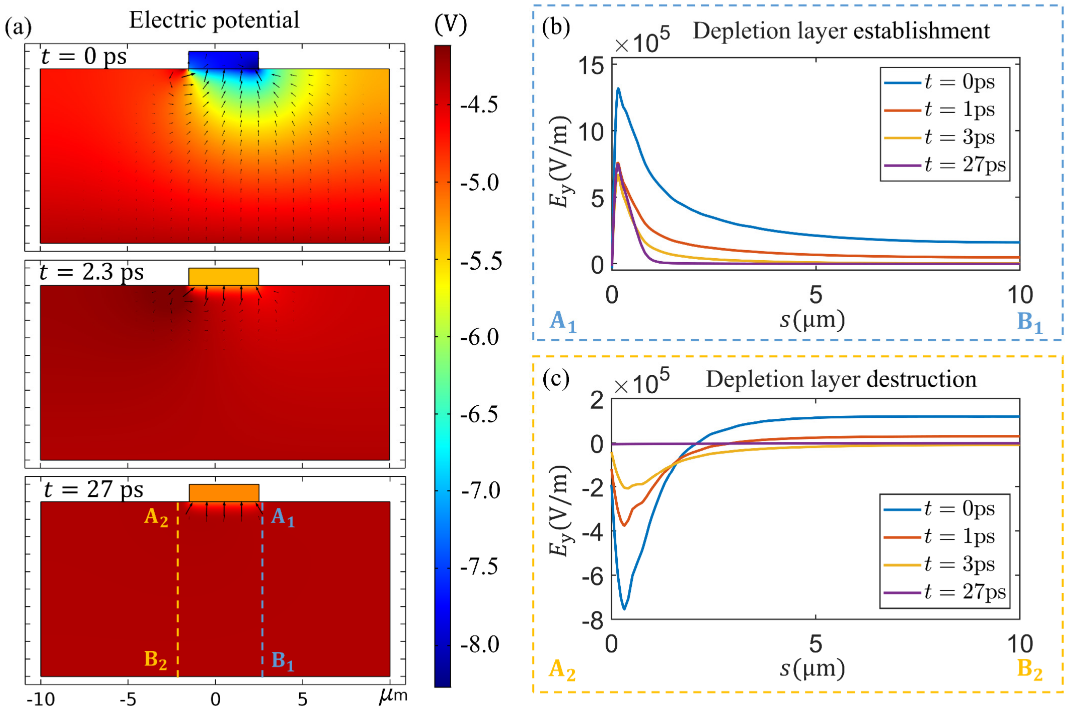

Supplementary Fig. 17 Electric potential and electric field distribution obtained by quasi-static simulation of the DLED mechanism when $\Delta x = 0.5$μm: (a) Relationship between the electric potential distribution over time, where the black arrow represents the direction of the electric field. (b) and (c) are the distribution of electric field in the y direction (upward is positive) E_y at the A_1B_1 ($x = 2.6$ μm, corresponding to the position of the depletion layer establishment) and A_2B_2 ($x = -2.1$ μm, corresponding to the position of the depletion layer destruction), respectively.

8.4 The estimation of unbalanced electron drift motion characteristic time

Based on the theory of static Schottky junction[5], we can obtain the charge of the depletion layer

$$Q_{\mathrm{D}} = eN_{\mathrm{D}}SL_{\mathrm{D}}, \tag{22}$$

where e is the charge of electron, $N_{\mathrm{D}} = 10^{15}\ \mathrm{cm}^{-3}$ is doping concentration of n-Si, $S = 4\ \mu\mathrm{m}^2$ is the contact area in simulation model and L_{D} is the depletion layer width. According to Eq. (15) and considering the bias voltage V_{b} applied to n-Si, we can obtain

$$L_{\mathrm{D}} = \sqrt{\frac{2\varepsilon_{\mathrm{r}}\varepsilon_0(V_{\mathrm{b}} + V_{\mathrm{D}})}{eN_{\mathrm{D}}}}, \tag{23}$$

where $\varepsilon_{\mathrm{r}} = 11.7$ is the relative permittivity of silicon, ε_0 is the vacuum permittivity and $V_{\mathrm{D}} = 0.87\ \mathrm{V}$ is the contact potential difference, which is obtained from the band diagram in Supplementary Fig. 15c. Substituting Eq. (23) into Eq. (22) can obtain

$$Q_{\mathrm{D}} = S\sqrt{2eN_{\mathrm{D}}\varepsilon_{\mathrm{r}}\varepsilon_0(V_{\mathrm{b}} + V_{\mathrm{D}})}. \tag{24}$$

It corresponds to the nature of capacitor, and the equivalent capacitance when $V_{\mathrm{b}} = 0$ is

$$C^{(\mathrm{eq})} = \frac{dQ_{\mathrm{D}}}{dV_{\mathrm{b}}}|_{V_{\mathrm{b}}=0} = S\sqrt{\frac{eN_{\mathrm{D}}\varepsilon_{\mathrm{r}}\varepsilon_0}{2V_{\mathrm{D}}}}. \tag{25}$$

The dynamic process of depletion layer establishment and destruction of the continuous sliding can be regarded as the charging and discharging process of the equivalent capacitor $C^{(\mathrm{eq})}$, so we can obtain the characteristic time with series resistance of $R = 10\ \mathrm{k\Omega}$ as

$$\tau = C^{(\mathrm{eq})}R = SR\sqrt{\frac{eN_{\mathrm{D}}\varepsilon_{\mathrm{r}}\varepsilon_0}{2V_{\mathrm{D}}}} = 3.9\ \mathrm{ps} \tag{26}$$

which is consistent with the characteristic time of current decay in Fig. 4b. It reflects the physical nature of simulation.

9. The discussion and classification of all reported ordinary S-G

We can divide the all reported ordinary Schottky generators (S-Gs) into nano tip[9,14] (NT S-Gs), macro tip[9,14-17] (MT S-Gs) and surface contact[18-23] (SC S-Gs) according to its contact area. The corresponding parameters of various type reported ordinary S-Gs plotted in Fig. 5 are shown in the Supplementary Table. 1. Among them, a typical NT S-G is consist of a conductive AFM tip pressed against a smooth surface, which will generate high pressure of 1-10 Gpa due to the small contact area of 10-100 nm^2 calculated by MDT model [10], which cause high friction stress to excite a large number of electrons leading to a high current density of 10^4-10^7 Am^{-2}. Therefore, NT S-Gs are likely based on the friction excitation mechanism [9,14]. Due to the small contact area, the highest total current achieved by NT S-Gs is observed to be around 0.15 nA [14], which is one order of magnitude smaller than S-SLG. Furthermore, the wear caused by high friction stress also makes the current of NT S-Gs completely decays during the first few cycles [14], which the lifetime is three orders of magnitude shorter than that of S-SLG.

The MT S-Gs has a similar structure to NT S-Gs. The difference is that the radius of macro conductive tip is generally around 1 mm, and the contact area are typically 0.1-1 mm^2 calculated by the Hertz model, which is much larger than NT S-Gs. Therefore, MT S-Gs has lower pressure of 50-670 Mpa and current density of 3-214 Am^{-2}.

The structure of SC S-Gs is different from that of NT-ordinary S-Gs and MT S-Gs, which is consist of a bulk metal material pressed against a smooth surface to form surface-to-surface contact, and the reported SC S-Gs are all in macroscale, so it has the larger contact area of 1-100 mm^2 and lower pressure of 2 kPa -6MPa compared to NT S-Gs and MT S-Gs, but the wear is still inevitable as the operation cycle increases [18,19], which indicates that the friction excitation mechanism cannot be excluded of reported SC S-Gs.

In conclusion, the above three types of ordinary S-Gs cannot achieve high current density and long lifetime simultaneously, that is, high current density means lower

lifetime, and vice versa. But we exclude the friction excitation mechanism in our S-SLG because the low friction energy is not enough to excite electrons, so it is based on a completely new mechanism, and most likely the DLED mechanism. Therefore, S-SLG is not limited by the above contradictory, and our experimental results shows that it achieved almost unlimited lifetime while maintaining high current density.

Supplementary Table 1 Parameters of different types of reported ordinary S-G[9,14-19,22,23]

Type	Structure	Contact area	Pressure	Average current	Current density (A/m^2)	Reported lifetime (s)	Ref
NT S-G	Pt/Ir-AFM tip/MoS2[14]	6-13 nm^2	0.83-2.31 GPa	0.08-0.15 nA	10^7	1-5	14
MT S-G	Metal tip/MoS2[14]	1500 μm^2	670 MPa	40 nA	27	14	14
	Stainless steel/p-Si[9]	1 mm^2	/	3 μA	3	2.5	9
	Al tip/ Perovskite[15]	0.03 mm^2	66.7 MPa	1.80 μA	60	10	15
	Phosphorus/ n-Si[16]	0.05 mm^2	160 MPa	6.2 μA	124	9	16
	N-Si/n-Si[17]	0.1 mm^2	50 MPa	21.4 μA	214	6	17
SC S-G	N-GaAs/p-Si[18]	1 mm^2	6 MPa	1.7 μA	1.7	3.2	18
	MoS2/AlN/ p-Si[18]	1 mm^2	5 MPa	1 μA	1	3600	18
	Graphene film/n-Si[19]	1 cm^2	50 KPa	3.3 μA	0.033	10000	19
	Al film/n-Si[19]	1 cm^2	50 KPa	4 μA	0.04	10000	19

	n-Si/p-Si[20]	1 cm^2	50 KPa	0.55 μA	0.0055	1200s	20
	Carbon Aerogel/Si[21]	0.785 cm^2	2.2 KPa	10 μA	0.13	20	21
	Metal/Si[22]	5 cm^2	36 KPa	18 μA	0.036	10	22
	MoS2/TiO2 /Ti[23]	1.7 mm^2	/	2 μA	1.2	6	23

References

1 Lu, X. K., Yu, M. F., Huang, H. & Ruoff, R. S. Tailoring graphite with the goal of achieving single sheets. *Nanotechnology* **10**, 269-272, doi:10.1088/0957-4484/10/3/308 (1999).

2 Zheng, Q. *et al.* Self-retracting motion of graphite microflakes. *Physical Review Letters* **100**, doi:10.1103/PhysRevLett.100.067205 (2008).

3 Wang, K., Qu, C., Wang, J., Quan, B. & Zheng, Q. Characterization of a Microscale Superlubric Graphite Interface. *Physical Review Letters* **125**, doi:10.1103/PhysRevLett.125.026101 (2020).

4 Cook, A. B. *et al.* Calibration of the scanning Kelvin probe force microscope under controlled environmental conditions. *Electrochimica Acta* **66**, 100-105, doi:10.1016/j.electacta.2012.01.054 (2012).

5 Zhang, Z. & Yates, J. T., Jr. Band Bending in Semiconductors: Chemical and Physical Consequences at Surfaces and Interfaces. *Chemical Reviews* **112**, 5520-5551, doi:10.1021/cr3000626 (2012).

6 Sader, J. E., Chon, J. W. M. & Mulvaney, P. Calibration of rectangular atomic force microscope cantilevers. *Review of Scientific Instruments* **70**, 3967-3969, doi:10.1063/1.1150021 (1999).

7 Sader, J. E., Larson, I., Mulvaney, P. & White, L. R. METHOD FOR THE CALIBRATION OF ATOMIC-FORCE MICROSCOPE CANTILEVERS. *Review of Scientific Instruments* **66**, 3789-3798, doi:10.1063/1.1145439 (1995).

8 Li, Q., Kim, K. S. & Rydberg, A. Lateral force calibration of an atomic force microscope with a diamagnetic levitation spring system. *Review of Scientific Instruments* **77**, doi:10.1063/1.2209953 (2006).

9 Liu, J. *et al.* Sustained electron tunneling at unbiased metal-insulator-semiconductor triboelectric contacts. *Nano Energy* **48**, 320-326, doi:10.1016/j.nanoen.2018.03.068 (2018).

10 Park, J. Y. & Salmeron, M. Fundamental Aspects of Energy Dissipation in Friction. *Chemical Reviews* **114**, 677-711, doi:10.1021/cr200431y (2014).

11 Wen, S. & Huang, P. *Principles of tribology*. (Wiley Online Library, 2012).

12 Johnson, K. L. & Johnson, K. L. *Contact mechanics*. (Cambridge university press, 1987).

13 Razavy, M. *Quantum theory of tunneling*. (World Scientific, 2003).

14 Liu, J. *et al.* Direct-current triboelectricity generation by a sliding Schottky nanocontact on MoS2 multilayers. *Nature Nanotechnology* **13**, 112-+, doi:10.1038/s41565-017-0019-5 (2018).

15 Hao, Z. *et al.* Co-harvesting Light and Mechanical Energy Based on Dynamic Metal/Perovskite Schottky Junction. *Matter* **1**, 639-649, doi:10.1016/j.matt.2019.05.003 (2019).

16 Lu, Y. *et al.* Tunable Dynamic Black Phosphorus/Insulator/Si Heterojunction Direct-Current Generator Based on the Hot Electron Transport. *Research* **2019**, doi:10.34133/2019/5832382 (2019).

17 Lu, Y. *et al.* Interfacial Built-In Electric Field-Driven Direct Current Generator Based on Dynamic Silicon Homojunction. *Research* **2020**, doi:10.34133/2020/5714754 (2020).
18 Lu, Y. *et al.* Direct-Current Generator Based on Dynamic PN Junctions with the Designed Voltage Output. *Iscience* **22**, 58-+, doi:10.1016/j.isci.2019.11.004 (2019).
19 Lin, S., Lu, Y., Feng, S., Hao, Z. & Yan, Y. A High Current Density Direct-Current Generator Based on a Moving van der Waals Schottky Diode. *Advanced Materials* **31**, doi:10.1002/adma.201804398 (2019).
20 Xu, R. *et al.* Direct current triboelectric cell by sliding an n-type semiconductor on a p-type semiconductor. *Nano Energy* **66**, doi:10.1016/j.nanoen.2019.104185 (2019).
21 Liu, U. *et al.* Tribo-Tunneling DC Generator with Carbon Aerogel/Silicon Multi-Nanocontacts. *Advanced Electronic Materials*, doi:10.1002/aelm.201900464 (2019).
22 Zhang, Z. *et al.* Tribovoltaic Effect on Metal-Semiconductor Interface for Direct-Current Low-Impedance Triboelectric Nanogenerators. *Adv. Energy Mater.* **10**, doi:10.1002/aenm.201903713 (2020).
23 Liu, J. *et al.* Scaled-up Direct-Current Generation in MoS2 Multilayer-Based Moving Heterojunctions. *Acs Applied Materials & Interfaces* **11**, 35404-35409, doi:10.1021/acsami.9b09851 (2019).
