# Supplementary material for: Microscale Schottky superlubric generator with high direct-current density and ultralong life
Source: Nat Commun. 2021 Apr 15;12:2268. doi: 10.1038/s41467-021-22371-1 (PMC8050059; doi:10.1038/s41467-021-22371-1)
Supplement: Supplementary file 3 — Description of Additional Supplementary Files [file 41467_2021_22371_MOESM3_ESM.docx]

**Description of Additional Supplementary Files**

File Name: Supplementary Movie 1

Experimental demonstration of Schottky superlubric generator (output current)
